# Culture in the Courtroom: Ethnocentrism and Juror Decision-Making

**DOI:** 10.1371/journal.pone.0137799

**Published:** 2015-09-09

**Authors:** Evelyn M. Maeder, Susan Yamamoto

**Affiliations:** 1 Institute of Criminology and Criminal Justice, Carleton University, Ottawa, Ontario, Canada; 2 Department of Psychology, Carleton University, Ottawa, Ontario, Canada; University of Western Brittany, FRANCE

## Abstract

The purpose of this study was to investigate whether a culturally-based argument in a non-insane automatism defense would be detrimental or beneficial to the defendant. We also examined how juror ethnocentrism might affect perceptions of such a defense. Participants read a fictional filicide homicide case in which the defendant claimed to have blacked out during the crime; we manipulated whether culture was used as an explanation for what precipitated the defendant’s blackout. We conducted path analyses to assess the role of ethnocentrism in predicting lower defendant credibility, and harsher verdict decisions. Results revealed an interaction between ethnocentrism and defense type, such that ethnocentrism related to lower perceived defendant credibility in the cultural condition, but not in the standard automatism condition. This study marks a starting point for empirically investigating the role of culture in the courtroom, which may aid scholars in discussing the merits of a standalone cultural defense.

## Introduction

In today’s growing global society, it is necessary to be aware of the ways in which culturally diverse individuals interact with the already complex legal system. As the 2010 Census illustrated, immigration trends indicate that cultural diversity is an increasing inevitability in the United States. For example, since the year 2000, the Asian-American population has grown at over four times the rate of the United States total population [1]. This rapidly developing multiculturalism likely results in diverse perspectives on what constitutes a morally justifiable, or at least explicable, act. The risks inherent in glossing over other cultural worldviews begin to manifest when considering the constitutional guarantee that a jury is composed of a defendant’s ‘peers’. In legal cases, jurors are tasked with evaluating a defendant’s actions while faced with conflicting accounts of an incident. However, defendants belonging to other cultures may present an explanation that is difficult to coalesce with the juror’s own worldview. Instead, as Dundes Renteln [2] argued, the viewpoint that jurors adopt may be that of the dominant culture. Persons enculturated in the United States—i.e., who have unconsciously adopted such a culturally dictated way of thinking—may have internalized those same values championed by the American legal system.

While a substantive “cultural defense” (i.e., in which a defendant asks the court to consider how his or her cultural background helps to justify or excuse the behavior in question) has not been established, strategies for considering cultural differences are available. Cultural evidence may be used to augment formal defenses (e.g., provocation [3,4]). However, if a culturally based defense is more likely to result in a poor outcome for the defendant, then there may be implications regarding its utility, and more radical consequences for a potential standalone cultural defense. As Dundes Renteln [2] asserted, it is common for people to reject notions of a cultural defense automatically, on the grounds that it would result in chaos. The purpose of this study was to investigate juror decision-making in a non-insane automatism case (in which the defendant claims that the act was involuntary due to loss of control of his or her body) and an identical case in which the defense also argued that cultural issues precipitated the defendant’s ‘automatic’ state.

### Culture and the Courts

While a rich assortment of scholars has undertaken to understand culture, it remains a complex construct. Van Broeck [5] defined culture as follows: “An intersubjectif [sic] system of symbols, which offer the human being an orientation toward others, the material world, him or herself and the non-human. This symbolic system has a cognitive as well as an evaluative function”. Van Broeck further emphasized the importance of viewing culture as an overarching system of thinking, whereby moral norms are only a subset of that system. Spanning a wide range of influences, cultural viewpoints may dictate a variety of thoughts and behaviors, from how we appropriately greet one another to what constitutes a just punishment. Dundes Renteln [2] essentially equates ‘culture’ with “a way of life”. In her discussion surrounding the need to implement a substantive cultural defense, Dundes Renteln maintained that the automatic manner in which we internalize cultural values is key to understanding such a defense. Notably, while the construct more broadly encompasses variables such as race, investigating the impact of culture offers a unique perspective. Several psycholegal studies have implicated the race of various trial parties as a variable of interest [6–8]. The study of culture likely enters into a distinct psychological mechanism in terms of the biases that may surface, yet this topic is surprisingly understudied in the courtroom. After all, not all persons belonging to a certain race share the same cultural values, and it is certainly possible for people of different races to converge on influential belief sets. Of course, it sometimes happens that a person has immigrated to a country many years prior, yet maintains strong ties with a home culture. Therefore, this paper offers a discussion of culturally, not necessarily racially, driven attitudes, in terms of the extent to which participants view the United States as superior to other countries.

Importantly, what is labeled as a ‘cultural offense’ does not simply depend on the legal sanctions of that culture, or on the group’s outright approval of the act, but can be a graded appreciation for the unique situation [5]. For example, People v. Kimura [9] elucidated the divergent meanings given to suicide in Japanese and American culture. Mrs. Kimura attempted to kill herself alongside her children in the cultural practice of parent-child suicide, termed ‘oyako shinju’; however, she was rescued and the children did not survive. While the killing of one’s children provokes moral outrage in American culture, the Japanese community felt that her actions were not simply evil (though not per se moral), but understandable given the circumstances. The fact that this sentiment resulted in thousands of community members petitioning for leniency illustrates the influence of culture in framing how we think about events that, at first blush, appear morally straightforward [2]. Van Broek [5] noted that while the Japanese government formally penalizes filicide, parent-child suicide may be viewed much as the tragedy of car accidents is viewed, as an inevitable fact of life.

Indeed, such cultural cases involving the death of vulnerable persons are among the most controversial, and may be so distressing as to prompt onlookers to distance themselves from the event. Iga [10] also illuminated the striking differences between attitudes toward parent-child suicide in Japan and the United States. Describing the strong sense of group goals over individual goals, feelings of responsibility, and emphasis on conformity associated with altruistic suicide, Iga [10] underscored the importance of family harmony in Japanese culture. With regard to the case of Mrs. Kimura [9], the stigma and hardships faced by divorcees in Japan is also at the forefront; not only may they be unwelcome back into their parents’ homes, but there are virtually no social services to aid them. Therefore, committing parent-child suicide is understood—though not condoned—in Japan. The phenomenon of attempted parent-child suicide within two cultures with different overall attitudes toward the act provides a point of comparison for the ability of jurors to tolerate a cultural perspective, and was therefore explored in the current study.

A number of scholars have hotly debated the ethical role of a true ‘cultural defense’ within both British and U.S. law. As some have pointed out, both systems have been hesitant to adopt an independent cultural defense, contemplating the balancing act of protecting diverse cultural perspectives and protection against assailants being ‘excused’ from certain crimes [11]. Not only has a myriad of psychological research demonstrated that people are more lenient toward those in their own group [12], but intuition also tells us that it is harder for people to trust unfamiliar customs.

By the same token, what constitutes a reasonable version of an event may vastly differ depending on culture. In the case of Kong Moua [13], he claimed to have performed the ritual of ‘marriage by capture’, leading to a charge of rape against him. It is no stretch of the imagination to see how it may be difficult for jurors who are unfamiliar with certain cultural practices, or who are resistant to moral norms in other cultures, to accept a defendant’s claim as plausible in a case such as this. Erber and Fiske [14] argued that people are more likely to focus on information that is consistent with their belief set, discarding incompatible information. Hastie and Pennington [15] further remarked that many cultures pass down moral codes through storytelling, and that some dispute resolutions also involve stories describing the proper conduct. Hence, jurors’ views of a morally defensible act may be driven by cultural moral codes. Such biases can be particularly disturbing to the juror decision-making process, given that jurors may first individually form evidence into a plausible explanation of the event.

Moreover, Volpp [16] reminded us that the U.S. court itself could be thought to prescribe a culture. Hence Volpp cautioned that a substantive ‘cultural defense’ might in some sense promote the depiction of immigrants or persons from minority cultures as an ‘out-group’ in relation to the U.S. This implies the potential for culture in the courtroom to place jurors in a frame of mind in which they psychologically distance themselves from the defendant.

### Ethnocentrism

The pervasive influence of one’s cultural worldview means that it can interfere with the ability to relate to the situations of others. Social identity theory [17] posits that prejudice against others occurs as a function of the degree to which one identifies with his or her group [18]. The notion of out-group severity has been examined with respect to a number of constructs such as race and ethnicity [6, 18]. Negy, Shreve, Jensen, and Uddin [18] found that for White and Hispanic participants, stronger identification with their ethnic identity was associated with negative views of ethnic out-group members. Remarking on the complexity of ethnic identity, Negy et al. underscored the contribution of ethnic practices, commitment, and evaluations relative to that group among other factors. If the U.S. court can be construed as subscribing to—perhaps epitomizing—a specific set of cultural norms, then persons who view the U.S. as superior to other countries might be particularly apt to discriminate against cultural defendants.

As Bizumic [19] noted, scholars have described the concept of ‘ethnocentrism’ dating back to at least to the 19^th^ century. Most notably, Gumplowicz observed the ways in which people tend to “glorify their own and nearest”, while derogating seemingly different or ‘foreign’ others [19]. Sumner [20] later popularized this concept, noting that people use their own group as a central reference point, against which all other groups are “scaled and rated”. Adorno, Frenkel-Brunswik, Levinson, and Sanford [21] underscored the problem of in-group and out-group categorizations as it related to anti-semitic attitudes. Using one of the first ethnocentrism scales, they demonstrated that such attitudes extended to several perceived out-groups, and were indicative of an overall tendency to make such a comparison with many other groups.

Taylor and Jaggi [22] furthered this line of reasoning by investigating the role of causal attributions in ethnocentrism. According to attribution theory, following a negative event, people tend to engage in ‘causal search’ in an attempt to ascribe a reason for that event [23]. These causal attributions then lead to a series of cognitive, affective, motivational, and behavioral consequences along the dimensions of: locus of causality (internal or external), stability (enduring or changing), and controllability (having an effect on the outcome). Using a sample of members of the Hindu faith, Taylor and Jaggi [22] illustrated that participants tended to use internal causal explanations when explaining positive events for another Hindu person, but tended to use external causal explanations for negative events. Conversely, participants tended to attribute negative events to internal causes when evaluating the actions of a Muslim person.

These findings suggest that when asked to assess the actions of others, group membership can play a role, a phenomenon that is likely problematic for jurors. Jurors are expected to fill in the gaps of different possible scenarios presented at trial; therefore, highly ethnocentric attitudes may act as an automatic barrier for fair evaluation of the evidence.

Not only are ethnocentric attitudes a cause for concern in rational decision-making, but also they are far-reaching. In fact, many scholars agree that ethnocentrism is a natural phenomenon that characterizes all people in some way, and that it is simply a matter of degree [24–26]. Neuliep, Hintz, and McCroskey [25] argued that ethnocentrism has a profound effect on communication, especially insofar as it affects beliefs about the source of information; in the absence of information about a source, people tend to imagine one [25, 27]. Neuliep and colleagues [25] demonstrated a negative relationship between ethnocentrism and perceived credibility of an out-group member who was being interviewed for a job. Overall, Neuliep et al. [25] found that ethnocentric attitudes were negatively related to perceived attractiveness, competence, and character. These findings indicate that ethnocentrism is a likely influence in juror decision-making, given that jurors’ evaluations and eventual decisions rest on such perceptions [28, 29]. Stephan and Stephan [30] examined two forms of ethnocentrism in juror decision-making among Hispanic and non-Hispanic college participants. They reasoned that not only can in-group membership offset potential ethnic bias in court, but also the language that the defendant uses during the trial can evoke bias. Specifically, they found that when a defendant’s testimony was translated as opposed to given in English, participants who were not part of the defendant’s ethnic group were more likely to vote guilty. They concluded that this effect was owing to both the tendency to view out-group members less favorably, as well as the belief among jurors that English should be used in U.S. courts rather than accommodating foreign languages. These two levels of ethnocentrism suggest that jurors may have explicit beliefs about the role of cultural diversity in court, as well as having automatic biases against such defendants.

It is necessary to grant that ethnocentrism is not wholly an undesirable trait [31]. As Neuliep and McCroskey [31] reminded us, while ethnocentrism can be dangerous, it is also related to valuable factors that contribute to group well-being, such as self-sacrifice for the collective. However, insofar as more extreme ethnocentrism results in discriminatory decision-making by jurors, it may be considered problematic. Indeed, Neuliep et al. [26] highlighted the dangers associated with a form of ethnocentrism that breeds perceived superiority, because it is intertwined with the application of power over those with contrary worldviews. Likewise, Dundes Renteln [2] questioned the logic of a “monolithic legal code”, asserting that the system itself may be a vehicle for ethnocentrism, wherein the dominant viewpoint is forced upon a country’s inhabitants. By way of example, Dundes Renteln [2] stressed the absurdity of asking an American bride to justify the custom of throwing a bouquet. If cultural dictates may cause a proclamation that even the mundane activity of driving may be done on the ‘wrong’ side of the road, it is troubling to imagine ethnocentric decision-making in an actual moral arena. A clash of moral viewpoints may be especially problematic when the defendant’s state of mind (relating to the voluntariness of the physical act) during the crime is at issue.

### The Automatism Defense

Automatism is a full defense in which the defendant claims that the act was involuntary, and that he or she was not conscious of his or her action. The automatism defense—which exists in Canada, the United Kingdom, and the United States—is used to offset the voluntariness of the alleged crime [32]. If a defense of sane automatism is successful, the defendant is acquitted.

There is no claim that the defendant did not commit the act in question, only that he or she did so in an automatic state. One of the most famous uses of the defense in Canada is R v. K. [33], in which a man killed his wife after hearing that she was going to leave him. Mr. K. reported embracing her and later realizing that she was dead; the defense successfully argued that he had received an ‘emotional blow’ akin to a physical blow to the head, in which he became out of control [34].

The U.S. case of People v. Wu [35] illustrated the partial success of a cultural automatism defense [2]. Helen Wu, a Chinese woman, argued that she strangled her son because she was sent into a fugue state, due to the shame of rejection from her lover [2]. However, after Wu was later granted a retrial, she was convicted of voluntary manslaughter. Importantly, the court remarked that there was no reason that the jury should not be instructed to consider Wu’s cultural background. The claim of such emotional shock thus allows for an automatism defense that is not necessarily physical in nature. An extreme upset provoked by the violation of specific cultural values may therefore be eligible for such a defense.

### The Current Study

Evidently, juror decision-making may be influenced by attitudes and expectations about moral norms, many of which are culturally defined. The current study sought to investigate differences in juror decision-making for a case in which cultural differences would be salient: a filicide case. If ethnocentric attitudes indeed relate to harsher treatment of culturally diverse defendants, then the use of cultural evidence toward an automatism plea may be counter-productive. Conversely, if cultural factors can provide the juror with useful evidence in considering the defendant’s mind set, then it may be an important tool for the fact finder. To assess how such factors might operate, we provided American participants with a fictional trial transcript (loosely based on People v. Kimura [9]) that described the case of a mother or father who claimed to have blacked out after learning of her or his spouse’s adultery and drove off of a bridge, resulting in the death of his or her two children. Given that we tested a filicide case, we varied the gender of the defendant, in the event that the killing of one’s child would be viewed as a greater violation for a mother versus a father. Comparing the treatment of men and women in filicide cases, Wilczynski [36] concluded that men who kill their children tended to be viewed as evil, but women who kill their children are viewed as ill. While we appreciate that gender may play a powerful role in other case types [37], gender was not per se a main variable of interest.

### Hypotheses

**Fig 1:**
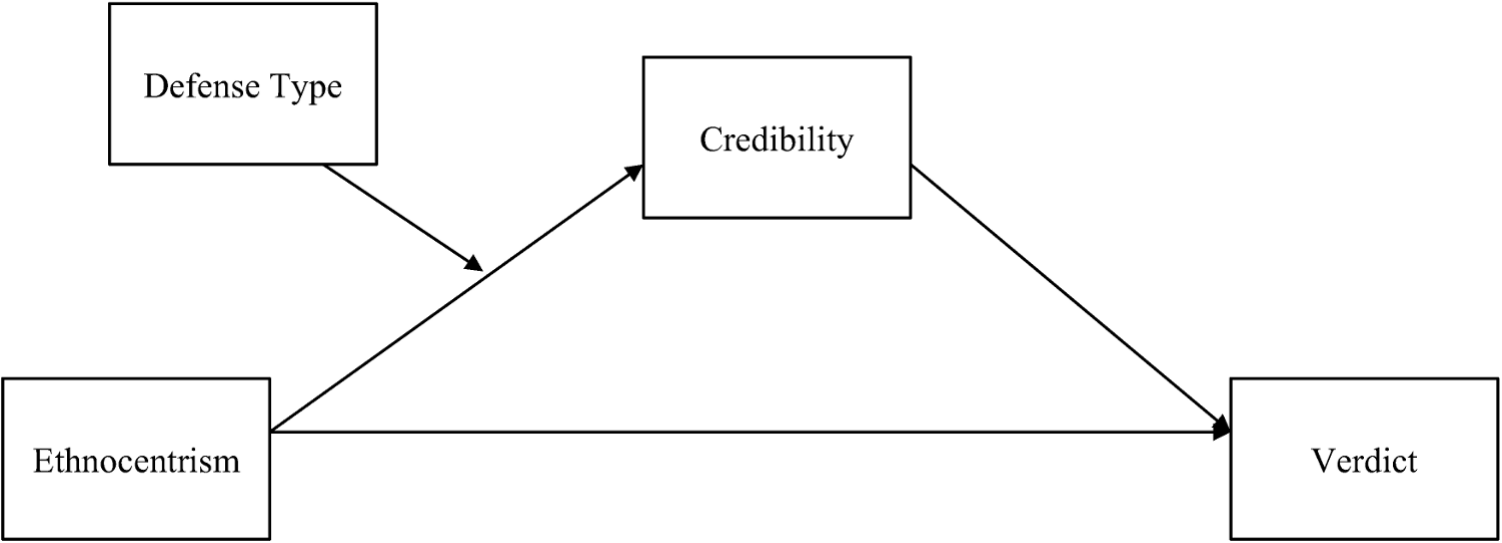
Hypothesized relationship between ethnocentrism and three-category verdict decision via credibility, with defense type moderating the association between ethnocentrism and credibility.

Researchers have found that jurors’ eventual decisions rest on their overall appraisal of the defendant [28, 29]. Further, as aforementioned, Stephan and Stephan [30] found that ethnocentrism could actually lead to more guilty verdicts, owing to beliefs about the appropriateness of foreign languages in court, as well as biases against out-group members. Therefore, it appears that ethnocentrism may lead not only to negative appraisal of a defendant, but also to discriminatory behavior. We sought to test a path model (see Fig 1) that would demonstrate predicted relationships between ethnocentrism, credibility, and eventual verdict decision, as well as whether this process might differ as a function of cultural evidence. To do so, we employed multiple group path analysis, which simultaneously tested credibility regressed on ethnocentrism, and verdict decision regressed on credibility and ethnocentrism for the cultural automatism and standard automatism conditions in two separate models.

We expected that ethnocentrism would significantly predict perceived defendant credibility, given the literature suggesting that higher ethnocentrism reduces credibility ratings for out-group members [25, 27], and that greater credibility would in turn predict greater likelihood of a more lenient verdict decision. We also hypothesized that cultural evidence would exacerbate the influence of ethnocentrism on perceived credibility, given that it might prompt jurors to other the defendant. While in the standard automatism condition there was no mention of Japanese culture, the names of the defendant and other trial parties were stereotypically Japanese (at least uncommon among American names), which was likely to produce some effect of ethnocentrism, albeit a weaker one than with the explicit mention of culture. In brief, we predicted indirect effects of ethnocentrism on three-category verdict decision via perceived defendant credibility, but also a significant difference in the magnitude of those effects as a function of cultural evidence.

## Method

### Participants

Participants were recruited via Amazon’s Mechanical Turk. They were screened for juror eligibility in the United States (i.e. U.S. citizens, 18 years of age or older, with no prior felony convictions for which they had not received a pardon) prior to analyses. We also screened out participants who did not attend sufficiently to the cultural defense manipulations (see materials section). Remaining participants’ ages ranged from 18 to 80 years old (*M* = 33.1 years, *SD* = 11.9). Men (*n* = 87) comprised 43.1% of the sample and women (*n* = 115) comprised 56.9% of the sample. The sample was 77.2% Caucasian, 8.9% Black/African-American, 7.4% Asian, 4% Latino/a, 1% American Indian,. 5% as Middle Eastern, and 1% identified as ‘other’. No participants reported affiliation with Japanese culture.

### Materials

#### Trial transcripts

Four fictional, eight page trial transcripts were created in which either a standard automatism defense or an automatism defense with a cultural component was presented; we also varied the gender of the defendant. The trial described a Japanese defendant charged with second-degree murder, who learned of his or her spouse’s infidelity when picking up their two children from school. The transcripts included opening arguments, as well as testimony from an eyewitness, the arresting officer, a psychiatrist, the defendant, and his or her spouse. It was described how after learning of his or her spouse’s affair, the defendant then drove the car over a bridge and into a lake, and how he or she was rescued while the children were unable to be saved. The prosecution argued that the defendant killed the two children in an attempt to punish his or her spouse. The defense argued that the defendant received a severe psychological blow owing to the adultery confession. All participants read expert testimony from Dr. Green, a psychiatrist, who described how the defendant was unable to control his or her body after feeling such intense shame, indicating that a fugue state had resulted.

In the cultural automatism condition, Dr. Green stated: “In Japanese culture, family always comes first, and inability to keep the family together is considered a grave failure. Given how serious divorce is in (her/his) culture, and the intense shame that resulted for (her/him), it is very plausible that (she/he) would be placed in such a dissociative state, unable to cope with the cultural implications of the divorce.” The defense attorney also reminded the jury that cultural issues were relevant to the case by stating “You have to remember that (Mrs./Mr.) Fujikawa’s culture is relevant in this case. In Japanese culture, divorce is a very serious issue, and it is thought to reflect failure on the part of the couple. Because of the implications of divorce in that culture, (Mrs./Mr.) Fujikawa was unable to cope with (her husband’s/his wife’s) words and so (she/he) blacked out.” The standard automatism defense transcript described the same parties of the trial, the key difference being that there was no explicit reference to culture made at any time. The defense simply argued that the defendant received an intense psychological blow from the news of the affair and blacked out: “You have to remember that to (Mr./Mrs.) Fujikawa, divorce is a very serious issue, and it is thought to reflect failure on the part of the couple.” In order to avoid confounding the role of expert testimony with the possible effects of cultural evidence toward the automatism plea, the psychiatrist appeared in both defense types, whose testimony differed among conditions only in that the reason for the ‘emotional upset’ was attributed to the seriousness of adultery in the defendant’s view rather than in the defendant’s culture: “She indicated that for her, family always comes first, and she considers divorce to mean grave failure”.

#### Dependent variables

Following the trial transcript, participants were asked to issue a verdict in the case: guilty of second-degree murder, guilty of manslaughter, or not guilty by reason of automatism. They also rated their perceptions of the defendant’s credibility (where *1 = not at all credible*, and *10 = very credible*).

#### Manipulation check

The manipulation check was conducted after participants had completed the trial questionnaire, and read: “Please indicate whether the following statement is true or false: the psychiatrist argued that traditional cultural values contributed to the defendant’s fugue state”. Participants chose from the options: true, false, or I don’t know. For the cultural automatism conditions, the failure rate was low, with nine and six participants failing for the male and female defendant conditions respectively. However, it seems that several participants falsely remembered a cultural argument in both standard automatism conditions. For the male, no culture condition, 87 participants chose “true”, while 25 participants chose “I don’t know”. For the female, no culture condition, 75 participants chose “true”, while 25 participants chose “I don’t know”. Implications are considered in the discussion section.

#### Questionnaires

Participants also completed Neuliep and McCroskey’s [31] United States ethnocentrism scale, which consists of 16 items measured on a 5-point Likert scale, assessing the extent to which participants believe that the United States is a superior country to other countries (e.g., “A lot of other countries are primitive compared to the United States”). The ethnocentrism measure yielded good internal consistency for this sample (α = .89). Participants also completed a modified version of the Cortes, Rogler, and Malgady [38] biculturalism scale, slightly reworded for use on a diverse ethnic sample, which consists of 20 items assessing how much the participant’s other culture is part of his or her life. It should be noted that we did not obtain enough responses to the biculturalism scale to justify meaningful analysis of the variable, as most participants did not identify as bicultural.

### Procedure

Participants were directed to a link via Mechanical Turk to the survey using Qualtrics, a type of online study software. After completing informed consent, participants were randomly assigned to a 2 (automatism: cultural vs. standard) by 2 (defendant gender: man vs. woman) design. Participants were instructed on the burden of proof, reasonable doubt, and the criteria for the automatism defense [39, 40]. Participants then provided a verdict decision, verdict confidence ratings, and completed measures of their perceptions of the defendant. The subsequent questionnaire section also contained an ‘attention check’ after the ethnocentrism scale, which stated: “How’s the weather today? Please select ‘sunny’, so that we know you have read the whole question”; use of this technique resulted in exclusion of 5 participants who answered the question incorrectly. Finally, participants were debriefed and provided with a completion code to receive compensation.

### Ethics statement

The Carleton University Psychology Research Ethics Board specifically approved this study (Reference # 13–088). After reading a recruitment notice and following the online survey link, participants completed an informed consent form. This form stated that participation was entirely voluntary, and at any point during the study, they had the right to not complete certain questions, or to withdraw without penalty. In order to proceed with the study, participants needed to select the option stating: “I consent to participate”. If participants selected “I do not consent to participate”, they were automatically directed to a debriefing form, and received a completion code to obtain compensation. Because deception was used (i.e., participants were only told that this study pertained to “how people process trial information”), at the end of the study participants read a debriefing form describing the rationale for the study, and the reason that deception was necessary. Following debriefing, participants completed a second consent form, which asked if they would allow the use of their data, or if they would prefer the data were deleted. We deleted all participant responses that indicated non-consent prior to analyses. The Carleton Psychology Research Ethics Board approved this consent procedure, and this project was completed within the guidelines set forth by the American Psychological Association.

## Results

In the standard automatism condition, 32.6% (*n* = 32) of participants selected a verdict of not guilty by reason of automatism, 48% (*n* = 47) selected manslaughter, and 19.4% (*n* = 19) selected second-degree murder. In the cultural automatism condition, 31% (*n* = 31) of participants selected a verdict of not guilty by reason of automatism, 49% (*n* = 49) selected manslaughter, and 20% (*n* = 20) selected second-degree murder. In order to determine the relationships between ethnocentrism, defense type, credibility, and verdict decisions, we conducted a multiple group path analysis using MPlus software [41] in which we compared the invariance of the paths between the cultural automatism and standard automatism conditions. We initially tested the possible impact of defendant gender via analyses of variance and hierarchical loglinear analysis, but found no main effects or interactions on the outcomes of defendant credibility and verdict. Therefore, we collapsed across those conditions. By using path analysis, we were able to assess potential differences in the process by which jurors reached a verdict when cultural evidence was present versus absent. Table 1 depicts descriptive statistics as well as bivariate relationships between the variables of interest within the two conditions.

**Table 1:** Descriptive statistics and correlations between variables.

**Standard Automatism Condition**					
	*M*	*SD*	Ethnocentrism	Credibility	Verdict
Ethnocentrism	2.7	.65	1		
Credibility	6.1	2.5	.16	1	
Verdict			-.13	-.53**	1
**Cultural Automatism Condition**					
	*M*	*SD*	Ethnocentrism	Credibility	Verdict
Ethnocentrism	2.7	.60	1		
Credibility	5.5	2.4	-.21*	1	
Verdict			.16	-.48**	1

* *p* < 05

**p <. 001

**Fig 2:**
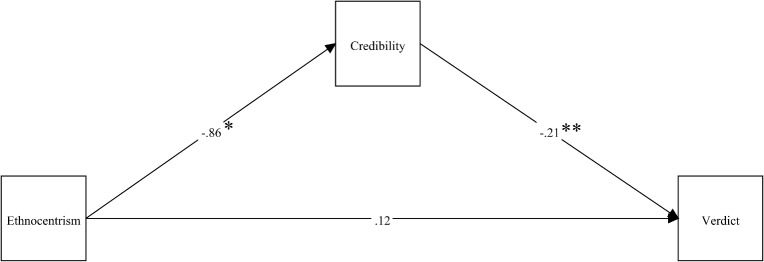
Path diagram depicting unstandardized relationships between ethnocentrism, defendant credibility, and three-category verdict for cultural automatism condition. Note: *p <. 05, **p <. 001.

Fig 2 depicts the path analysis for the cultural automatism condition, with credibility regressed on ethnocentrism, and three-category verdict regressed on credibility and ethnocentrism. Unstandardized regression coefficients are displayed.

Contrary to our hypothesis, ethnocentrism did not emerge as a significant predictor of three-category verdict decision for the cultural automatism condition, *B* = .12, *SE* = .17, *z* = .63, *p* = .53. In support of hypotheses, ethnocentrism significantly predicted perceived defendant credibility, *B* = -.86, *SE* = .38, *z* = -2.25, *p* = .03, such that higher ethnocentrism was associated with lower perceived credibility of the defendant. In support of hypotheses, credibility in turn significantly predicted verdict decision, *B* = -.21, *SE* = .02, *z* = -12.16, *p* <. 001, such that lower perceived credibility was associated with greater likelihood of a harsher verdict. The indirect effect of ethnocentrism on three-category verdict via perceived defendant credibility was significant, *B* = .18, *SE* = .09, *z* = 2.12, *p* = .03. Ethnocentrism accounted for only a small proportion of the variance, with an adjusted R^2^ of. 03 (p <. 05).

**Fig 3:**
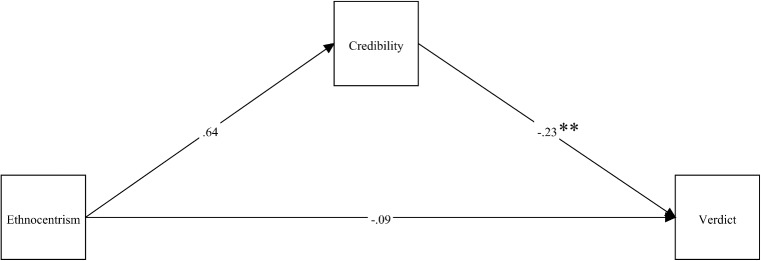
Path diagram depicting unstandardized relationships between ethnocentrism, defendant credibility, and three-category verdict for standard automatism condition. Note: **p <. 001.

Fig 3 depicts the same analyses for the standard automatism condition, which MPlus executes simultaneously. We were therefore able to uncover any statistically significant differences in the pathways as a function of defense type, by means of a Wald chi-square parameter constraint test for each hypothesized path.

As the non-significance of the pathways in Fig 3 clearly demonstrates, ethnocentrism does not have the same impact on credibility in the absence of cultural evidence. Contrary to hypotheses, ethnocentrism did not significantly predict either credibility, *B* = .64, *SE* = .39, *z* = 1.65, *p* = .10, or three-category verdict, *B* = -.09, *SE* = .16, *z* = -.56, *p* = .57. Accordingly, in the standard automatism condition there was no indirect effect, *B* = -.15, *SE* = .09, *z* = 1.61, *p* = .11. Comparing the indirect effects demonstrated a statistically significant difference, *z* = 2.62, *p* = .009. Unsurprisingly, the Wald test confirmed the need for a model in which parameters vary as a function of defense type with respect to the relationship between ethnocentrism and credibility, *χ* ^*2*^(1) = 7.56, *p* = .006. Hence the data show evidence of moderated mediation for the path between the independent variable and mediator variable, where ethnocentrism does not appear to impact on credibility unless cultural evidence is presented. As would be expected, credibility still showed the same significant relationship with three-category verdict, *B* = -.23, *SE* = .02, *z* = -10.69, *p* <. 001, such that lower credibility predicted greater likelihood of a harsher verdict. The tests for invariance of the pathways between the two groups with respect to a direct effect of ethnocentrism on verdict, as well as credibility predicting verdict, were non-significant. Therefore, there is no difference between the two groups in terms of those relationships.

Finally, we wished to test for any overall differences regarding the two outcome variables (i.e., defendant credibility and continuous verdict). An independent samples t-tests revealed that the cultural automatism (*M* = 5.5, *SD* = 2.4) and standard automatism conditions (*M* = 6.1, *SD* = 2.5) did not differ significantly in terms of perceived defendant credibility, *t*(196) = 1.63, *p* = .10, *Cohen’s d* = .23, 95% CI [-.11,. 57]. A chi-square test for independence revealed that the difference in verdict breakdown between the two conditions was non-significant, *χ* ^*2*^(2) = .06, *p* = .97.

## Discussion

The purpose of this study was to create an empirical foundation upon which to understand the role of culture in the courtroom. Since culture can significantly shape one’s worldview, the legal assumption that jurors can simply use their own cultural mindset may be problematic when they must take a divergent viewpoint. In particular, this study tested whether using culture to augment a formal legal defense would be detrimental to the defendant, owing to the possibility of ethnocentric attitudes. Using path analysis, we were able to investigate the juror decision-making process with respect to ethnocentrism as well as defense type, assessing how such factors relate to perceived defendant credibility and verdict decision. Results revealed that ethnocentrism is negatively related to credibility, which in turn relates to harsher verdict decisions, but only in the presence of cultural evidence. Hence, it does appear that cultural evidence played into how jurors evaluated the case, as a function of how much they viewed U.S. culture as ‘right’.

These findings seem to suggest that cultural evidence may work against the defendant if jurors are ethnocentric, rather than to aid the fact-finder in contemplating the evidence. It appears that when culture was invoked as a precipitating factor for the defendant’s blackout, higher ethnocentrism related to diminished defendant credibility, which may have served to undermine his or her story. As some scholars have expressed in regards to a substantive cultural defense, fears surrounding its misuse in favor of the cultural defendant are not the only cause for concern. As Volpp [42] contended, beliefs about the ‘backward’ nature of another culture may lead to a type of cultural essentialism, in which all such people are seen as bound by such customs. The results of this study demonstrate that the effect of ethnocentrism on defendant credibility may be key to the jurors’ judgments when considerations about such cultural ‘wrongness’ are particularly salient. Bias in favor of one’s culture is not an appropriate tool for the juror in evaluating the defendant’s claim; rather, it likely interferes with other important deciding factors, such as testimony that corroborates the claim.

It bears mentioning that we did not observe any defendant gender effects. We manipulated defendant gender in the event that there was general leniency toward a woman owing to the tendency to view women as ‘ill’ rather than as ‘evil’ [36]. It was also a possibility that, given the rareness of homicide trials with female defendants, participants would simply be more willing to consider contextual factors in such a case. However, recent meta-analytic work by Devine and Caughlin [37] found that in samples involving community participants, female defendants were convicted more often than male defendants. In general, they argued that the stereotype associating men with crime might not be as strong as it was since Mazzella and Feingold’s [43] influential meta-analysis (i.e., that the overrepresentation of males among offenders leads to harshness against them in court). Devine and Caughlin [37] noted that such gender differences likely vary as a function of case type. It is possible that differences would emerge in crimes for which gender is a more salient issue (e.g. spousal violence), especially where the incident involves more direct physical power. In any case, more research is needed to identify cases in which gender would impact on juror decisions, as scholars have discussed the complex interplay between multiculturalism and gender issues in court [42, 44].

### Strengths and Limitations

This study represents a first step in a program of research aimed at uncovering the issues surrounding culture in the courtroom. There are a number of reasons to be confident in the implications of these findings. First, we assessed a community rather than a student sample, lending more support to the ecological validity of the findings. Additionally, an attention check was used to ensure higher data quality, given that collection took place online rather than in a lab. Second, it is clear that the effects associated with the manipulations were owing to the cultural evidence rather than simply the ethnicity of the defendant, because the defendant had a Japanese name in both cases. Finally, the lack of research investigating the important psychological functions relating to culture in the courtroom is surprising; therefore, this study adds to the debate about the dangers of a standalone cultural defense, and provides a starting point for other researchers to contribute to the discussion.

As this study is among the first to investigate the topic, it is important to note the limitations that warrant caution in interpreting these findings. First, there was a high manipulation check failure rate among participants in the standard automatism conditions, such that most falsely remembered the psychiatrist as having argued that traditional cultural values contributed to the defendant’s automatic state. Notably, these findings do not imply an insufficiently salient manipulation of the cultural argument—rather, they suggest that the mere presence of a Japanese surname will lead some participants to infer a cultural argument where none is made. Almost all participants who were exposed to a cultural argument noticed the argument and answered the manipulation check correctly, suggesting that our findings cannot be attributed to a lack of attention to the cultural argument. What is troubling is that participants who read no cultural argument assumed that it must have been made, simply because the defendant had a Japanese surname. While we recognize that the large number of failures in only the standard automatism conditions is substantive data in and of itself, participants who extrapolated cultural relevance when it was not explicitly mentioned likely belong to a different population than those who answered the question correctly, and so we did not include them in analyses. Future researchers may wish to compare such decision including a White defendant condition to explore the role of ethnicity.

This study represents only one of many possible uses of culture to augment a defense. Therefore, results cannot be generalized to other plea types, such as the insanity defense or provocation. While the use of culturally based defenses may be rare, when successful they can be highly significant. For instance, People v. Chen [45] produced concerns that the lenient sentence Chen received for killing his wife supported violence against women [46]. In considering the plethora of ethnocentrism research demonstrating the permeating influence of culture, we find it likely that other case types may follow this pattern, and this study provides a launching point for other such investigations. The findings arising from this study are important, as we have shown that biases can become problematic in the mere reference of culture. Although there is no standalone cultural defense, the use of cultural evidence in court spans a wide array of cases [2], and there is risk of pervasive ethnocentric attitudes interfering when culture is on the table.

However, it is likely that there are other explanatory variables involved. For example, future researchers should consider the role of variables such as juror biculturalism. Because most participants did not identify as bicultural, we were unable to meaningfully examine the influence of experience adjusting to a second culture. Our sample was also composed mainly of White jurors, and we did not delve into the potential effects of race. Accordingly, the lack of a more nuanced investigation of subcultures also presents a limitation. Future researchers may wish to study the combined effects of culture with other notable variables, such as race, socioeconomic status, and geographic location. Finally, it remains to be seen how characteristics of the victim may affect juror decisions, for example, if the victim were of a different culture than the defendant; in this case, the victims and defendant were presented as sharing the same culture.

### Future Directions

It would be informative to further explore whether ‘ethnocentrism’ might show a different form when jurors are considering an insanity plea. After all, treating a cultural practice or explanation as a pathology is itself a type of ethnocentrism. Contrariwise, as Huckerby [47] illustrated, there is another way in which ‘othering’ is accomplished—based on the culture of the defendant—when a person is confronted with a threatening event. Examining the media documentation of the cases of Andrea Yates and Khoua Her, Huckerby [47] argued that views about violations of motherhood are different for ethnic mothers. More specifically, when confronted with the ultimate violation of parenthood, the killing of one’s child, explanatory labels of “bad” as opposed to “mad” are often sought when the defendant is an ethnic parent. As researchers have shown [19, 20], we tend to use our own group as the gold standard of appropriate behavior—any deviations from this standard may be viewed as ‘illness’, or they may be viewed as ‘evil’. Future researchers could investigate how ethnocentrism manifests in a cultural insanity plea.

### Conclusion

In sum, this study is the first step in a program of research that asks the question: how does the use of culture in a defense impact on trial outcomes? While legal scholars have discussed in great depth what role culture should play in the courtroom, empirical evidence can aid in uncovering whether concerns about cultural bias and the psychological consequences of a standalone defense are warranted. The preliminary evidence of this study suggests that ethnocentric attitudes may have a negative effect on juror decision-making when culture is used to explain the defendant’s state. However, it is noteworthy that this is not the result of the nature of a cultural defense in and of itself; rather, it is likely a complex interplay between different attitudes. It is imperative that researchers aid in developing a thorough understanding of the merits and dangers of a cultural defense by contributing a strong empirical foundation.

## Supporting Information

S1 Dataset (SAV)

S1 Text Female defendant trial transcripts. (PDF)

S2 Text Male defendant trial transcripts. (PDF)
