# Supplementary material for: Culture in the Courtroom: Ethnocentrism and Juror Decision-Making
Source: PLoS One. 2015 Sep 9;10(9):e0137799. doi: 10.1371/journal.pone.0137799 (PMC4564170; doi:10.1371/journal.pone.0137799)
Supplement: S1 Text — (PDF) [file pone.0137799.s002.pdf]

Female Defendant Trial Transcripts

Note: italics have been added to this document for emphasis of manipulated content, but were not italicized for participants

[BOTH CONDITIONS]
Background Information

Alleged Crime: Second Degree Murder
Victims: Mari Fujikawa, Kenichi Fujikawa
Defendant: Mrs. Yuriko Fujikawa D.O.B.: July 12th, 1979
Arrested: May 16th, 2012

Prosecution Opening Statement

The Prosecution will prove that Yuriko Fujikawa drove her two children into a lake intending to kill them. You will see that Mrs. Fujikawa received news that her husband had an affair and was leaving her, and wanted to punish him in the only way that she knew how, by taking away his children forever. Even though the defendant herself went into the water with the children, she committed second-degree murder when she drove off that bridge knowing full well that the children would die. The Prosecution will provide a witness that saw Yuriko Fujikawa drive into the lake that afternoon, show that she had motive to kill her children, and demonstrate that she was fully aware of her actions at the time of the crime. In short, we will prove beyond any reasonable doubt that Yuriko Fujikawa is guilty of murder in the second degree. Yuriko Fujikawa is a cold-blooded killer who took two innocent lives because she wanted revenge on her husband, plain and simple.

Defense's Opening Statement

The Prosecution is going to try to spin a heartless tale to make you believe that my client is evil. They are going to try to piece together a questionable story that does not fit with my client's great love of her children, but instead use some pretty slippery arguments to convince you that she is a bad person. The problem with the Prosecution's story is that my client is not evil; she is a mother who loved her family so much that when infidelity threatened to break it apart, she lost herself completely and blacked out. The Defense will show that the Prosecution's claim that my client meant to hurt those children she loved most out of anything in the world is baseless, and in fact it was that love of family that led to the severe psychological blow she suffered from hearing about her husband's affair. You will see that she did not commit this act voluntarily but instead lost control in those brief moments. Yuriko Fujikawa is not a calculating killer, and you, the jury, will be the real villains if you send this woman to jail.

Prosecution Witness, Andrew Morris
[BOTH CONDITIONS]

Prosecutor: What did you do on the afternoon of May 16th, 2012?
Morris: I went fishing at the lake.

Prosecutor: What did you see while you were at the lake?
Morris: I saw a car drive on to the bridge and start turning toward the edge. I didn't understand what they were doing, but then I saw the car go right off the bridge into the water.

Prosecutor: What did you do then?
Morris: I grabbed my cell phone and called 911, and asked them to send an ambulance and the police.

Prosecutor: Then what happened?
Morris: I had to try to help them, so I swam up to the front of the car, and managed to pull the woman in the driver's seat out through the window. I brought her to the shore and she seemed to be alright.

Prosecutor: Did she say anything?
Morris: She kept saying her kids were in the car.

Prosecutor: Then what did you do?
Morris: I swam as fast as I could back to the car to help the children

Prosecutor: What happened next?
Morris: The police arrived and jumped in the water to help. We were able to pull the two children ashore.

Defense Cross-Examination:

Defense: Mr. Morris, did you ask my client if anyone else was in the car?
Morris: No, she just kept saying it when we got to the shore.

Defense: Did she say anything when you pulled her out of the car?
Morris: It all happened so fast and it was hard to hear anything while I was trying to keep us above water.

Defense: Is it possible my client was asking you to help her children first?
Morris: Sure, it's possible.

Defense: Did Mrs. Fujikawa seem coherent to you?
Morris: She seemed very distraught and confused.

Prosecution Re-direct:

Prosecutor: So, you pulled Mrs. Fujikawa to the shore and just moments after the incident she was coherent enough to ask you to help her children?
Morris: Yes, I guess that's right. At least, I understood what she was saying.

Prosecution Witness, Officer Scott Townsend
[BOTH CONDITIONS]

Prosecutor: Can you please state your name and occupation?
Townsend: My name is Officer Scott Townsend, I am a police officer and was the first to arrive on scene on the day in question. I was also the officer that arrested Yuriko Fujikawa.

Prosecutor: What happened when you arrived on scene?
Townsend: When I arrived, I observed a woman lying on the shore, and a man in the water struggling.

Prosecutor: Who did you identify the woman on shore as?
Townsend: The defendant, Yuriko Fujikawa.

Prosecutor: What did you do then?
Townsend: I got into the water to help the man. We were able to pull the two children to the shore. At that time the paramedics attempted to resuscitate the children and transported them to the hospital.

Prosecutor: What happened after that?
Townsend: While the paramedics were attending to Mrs. Fujikawa, I interviewed the witness, Andrew Morris.

Prosecutor: What did you find out from interviewing Mr. Morris?
Townsend: He indicated that he had seen the car drive off of the bridge into the water, and that the incident did not appear to be a simple car accident. At that time, since the children were in critical condition and there was evidence to suggest that she purposefully drove off of the bridge, not as a result of loss of control of the vehicle, I arrested Mrs. Fujikawa.

Defense Cross-examination:

Defense: Officer Townsend, did you interview my client at the time of the event in question?
Townsend: Yes, I did.

Defense: And would you say that she was able to answer your questions?
Townsend: Mrs. Fujikawa acted strangely and was not able to answer my questions coherently.

Prosecution Re-direct:

Prosecutor: Officer Townsend, did the defendant communicate to you at any time that she was aware that her children were in critical condition?
Townsend: She continued to ask about her children, if they were alive.

Prosecutor: So, just moments after the incident, when she was supposedly unaware of what was happening, she communicated that she knew the children were in danger, and that they might not live?
Townsend: Yes, that's correct.

Prosecution Witness, Mr. Toshi Fujikawa
[BOTH CONDITIONS]

Prosecutor: Could you please state your name and relationship to the defendant?
Mr. Fujikawa: My name is Mr. Toshi Fujikawa; I am married to Yuriko Fujikawa.

Prosecutor: Did you speak to the defendant on the day in question?
Mr. Fujikawa: I called her that afternoon when she was picking the kids up from school.

Prosecutor: Why did you call your wife that afternoon?
Mr. Fujikawa: I called to tell her that I could no longer stay in this marriage. I could no longer keep it a secret that I love another woman. I planned to leave before she and the kids came home.

Prosecutor: How did she react to this news?
Mr. Fujikawa: She sounded very angry. We had been fighting for some weeks before that conversation, and she would yell and curse at me every time.

Prosecutor: Can you recall what the defendant said?
Mr. Fujikawa: She said something like "you'll regret this."

Prosecutor: How long did your conversation last on that particular occasion?
Mr. Fujikawa: Only a few minutes, I knew if I stayed on the phone longer she would only yell and I had already made up my mind. So I hung up after just a few minutes.

Defense Cross-examination:

Defense: Mr. Fujikawa, did you often pick the children up from school?
Mr. Fujikawa: Yuriko usually did that.

Defense: Would you say that your wife spent a lot of time with the children, took good care of them?
Mr. Fujikawa: Yes, she did put a lot of time in to taking care of them.

Defense: And in all the times you claim to have fought with your wife, at any time did you observe her to take her anger out on the children?
Mr. Fujikawa: No, she only got angry with me.

Defense Witness, Dr. Elliot Green
[CULTURAL AUTOMATISM CONDITION ONLY]

Defense: Can you please state your name and occupation for the court?
Green: Yes. My name is Dr. Elliot Green, I am a psychiatrist working at the Health Science Center.

Defense: And what are your credentials?
Green: I have a medical degree and have been practicing psychiatry for 20 years now.

Defense: Have you spoken extensively with my client, Mrs. Fujikawa?
Green: Yes. I have been meeting with Mrs. Fujikawa once a week for the past month. We have discussed the incident in a lot of detail.

Defense: Could you describe to the court what you learned from meeting with Mrs. Fujikawa?
Green: Certainly. She had communicated to me that she began to suspect her husband was being unfaithful, and that she felt very distraught over that. She indicated that family is the most important thing in her life and that she could not cope if she did not have them.

Defense: And what about the day in question, did the two of you speak about that?
Green: Yes, we did. She described getting in to the car and blacking out after hearing her husband's words. The next memory she reported was feeling very disoriented surrounded by police officers. She remarked to me that at that time, she feared for her children.

Defense: Could you describe to the court what can happen when a person experiences a psychological trauma?
Green: Yes. Sometimes, when the mind receives information for which it is unprepared, a condition can occur in which there is loss of the usual integration of personal identity and memories, sensory and motor function. As such, there is a splitting of specific mental activities from the rest of conscious awareness. In less extreme cases, for example, we may drive to a destination while distracted, and not recall the actual drive.

Defense: In your opinion, is Mrs. Fujikawa's experience consistent with such a state?
Green: Yes. Receiving such news as she did that her husband would be leaving would be much the same as receiving a physical blow to the head, except that it was a blow to the mind. Receiving such emotional news can leave one in a fugue state for several minutes, where continuing to drive is a very plausible event. *In Japanese culture, divorce is a very*

serious issue. Therefore, a person from her culture will typically react quite strongly to such news.

Defense: Could you elaborate on what kind of experience could prompt such a severe reaction?
Green: Mrs. Fujikawa talked at length about how much her family was central to her life. She often spoke of her willingness to do anything for the sake of family. *In Japanese culture, family always comes first, and inability to keep the family together is considered a grave failure. Given how serious divorce is in her culture, and the intense shame that resulted for her, it is very plausible that she would be placed in such a dissociative state, unable to cope with the cultural implications of the divorce.* She would not likely receive support from extended family in this case, and would not necessarily be aware of social support programs here.

Defense: In your opinion, is Mrs. Fujikawa trying to mislead you into believing she experienced this trauma?
Green: No, I do not believe so. In my 20 years of practice, I have come into contact with a few cases of false claims of mental trauma and loss of control. Typically, such a person would have inconsistencies in their report or might provide unnecessary details of the event.

Prosecution Cross-examination:
Prosecutor: Dr. Green, is the area of dissociative states your area of expertise?
Green: No, I mostly treat patients with severe long-term mental disorders.

Prosecutor: Are you an expert in deception, Dr. Green?
Green: No, I am not. But I have many years experience treating real psychological issues.

Prosecutor: I see. You said that Mrs. Fujikawa remarked that she was suspicious of her husband's infidelity in the time leading up to the crime.
Green: Yes, that's correct.

Prosecutor: So, isn't it possible that Mrs. Fujikawa was very angry at her husband as a result?
Green: Yes, that's possible. But from my interviews with Mrs. Fujikawa, I believe this saddened her more than anything.

Prosecutor: To your knowledge, had Mrs. Fujikawa suffered a fugue state like this prior to the incident?

Green: No, not to my knowledge. This incident seemed to be particularly upsetting to her so as to create the dissociative state.

Prosecutor: So, no prior fugue states such as this, and she just happens to experience one when she breaks the law?
Green: She did not indicate she had suffered something like this before.

Prosecutor: Well that sounds pretty convenient for Mrs. Fujikawa, doesn't it. So, to clarify, you do not typically treat patients who have had fugue states, and this was Mrs. Fujikawa's first ever fugue state.
Green: I suppose that's true, yes.

Defense Witness, Dr. Elliot Green
[STANDARD AUTOMATISM CONDITION ONLY]

Defense: Can you please state your name and occupation for the court?
Green: Yes. My name is Dr. Elliot Green, I am a psychiatrist working at the Health Science Center.

Defense: And what are your credentials?
Green: I have a medical degree and have been practicing psychiatry for 20 years now.

Defense: Have you spoken extensively with my client, Mrs. Fujikawa?
Green: Yes. I have been meeting with Mrs. Fujikawa once a week for the past month. We have discussed the incident in a lot of detail.

Defense: Could you describe to the court what you learned from meeting with Mrs. Fujikawa?
Green: Certainly. She had communicated to me that she began to suspect her husband was being unfaithful, and that she felt very distraught over that. She indicated that family is the most important thing in her life and that she could not cope if she did not have them.

Defense: And what about the day in question, did the two of you speak about that?
Green: Yes, we did. She described getting in to the car and blacking out after hearing her husband's words. The next memory she reported was feeling very disoriented surrounded by police officers. She remarked to me that at that time, she feared for her children.

Defense: Could you describe to the court what can happen when a person experiences a psychological trauma?
Green: Yes. Sometimes, when the mind receives information for which it is unprepared, a condition can occur in which there is loss of the usual integration of personal identity and memories, sensory and motor function. As such, there is a splitting of specific mental activities from the rest of conscious awareness. In less extreme cases, for example, we may drive to a destination while distracted, and not recall the actual drive.

Defense: In your opinion, is Mrs. Fujikawa's experience consistent with such a state?
Green: Yes. Receiving such news as she did that her husband would be leaving would be much the same as receiving a physical blow to the head, except that it was a blow to the mind. Receiving such emotional news can leave one in a fugue state for several minutes, where continuing to drive is a very plausible event. *In Mrs. Fujikawa's case, divorce*

seemed to her a very serious issue. Therefore, she likely reacted quite strongly to her husband's news.

Defense: Could you elaborate on what kind of experience could prompt such a severe reaction?
Green: Mrs. Fujikawa talked at length about how much her family was central to her life. She often spoke of her willingness to do anything for the sake of family. *She indicated that for her, family always comes first, and she considers divorce to mean grave failure. Given how serious divorce is for her*, it is quite plausible that she would be placed in such a dissociative state, unable to cope with the implications of the divorce. She did not expect to receive support from extended family, and did not indicate she was aware of any social support programs.

Defense: In your opinion, is Mrs. Fujikawa trying to mislead you into believing she experienced this trauma?
Green: No, I do not believe so. In my 20 years of practice, I have come into contact with a few cases of false claims of mental trauma and loss of control. Typically, such a person would have inconsistencies in their report or might provide unnecessary details of the event.

Prosecution Cross-examination:
Prosecutor: Dr. Green, is the area of dissociative states your area of expertise?
Green: No, I mostly treat patients with severe long-term mental disorders.

Prosecutor: Are you an expert in deception, Dr. Green?
Green: No, I am not. But I have many years experience treating real psychological issues.

Prosecutor: I see. You said that Mrs. Fujikawa remarked that she was suspicious of her husband's infidelity in the time leading up to the crime.
Green: Yes, that's correct.

Prosecutor: So, isn't it possible that Mrs. Fujikawa was very angry at her husband as a result?
Green: Yes, that's possible. But from my interviews with Mrs. Fujikawa, I believe this saddened her more than anything.

Prosecutor: To your knowledge, had Mrs. Fujikawa suffered a fugue state like this prior to the incident?

Green: No, not to my knowledge. This incident seemed to be particularly upsetting to her so as to create the dissociative state.

Prosecutor: So, no prior fugue states such as this, and she just happens to experience one when she breaks the law?
Green: She did not indicate she had suffered something like this before.

Prosecutor: Well that sounds pretty convenient for Mrs. Fujikawa, doesn't it. So, to clarify, you do not typically treat patients who have had fugue states, and this was Mrs. Fujikawa's first ever fugue state.
Green: I suppose that's true, yes.

Defense Witness, Yuriko Fujikawa
[BOTH CONDITIONS]

Defense: What happened on the afternoon of May 16th?
Mrs. Fujikawa: I picked up my children from school. My husband called me as I was helping them into the car.

Defense: Were you happy to see them?
Mrs. Fujikawa: Yes of course. They were happy to see me too. I picked them up every day, and it was always the best part of my day.

Defense: I know this is very difficult, but can you tell the court what your husband said when you spoke with him?
Mrs. Fujikawa: I think he said…that he would be leaving that day. Leaving the family.

Defense: How did that make you feel, when he told you that?
Mrs. Fujikawa: It was very difficult to hear. My family is my life. Family is all that matters. To abandon your family, it's unthinkable.

Defense: I know how much you loved your children, Mrs. Fujikawa**.** Can you try to describe what happened after that?
Mrs. Fujikawa: No, I can't. I just can't quite understand what happened. I was talking with my husband, and the next thing I knew I was struggling to breathe and there were people all around me.

Defense: What else can you recall from the drive home?
Mrs. Fujikawa: I don't know. I felt sick, I don't know what happened. I think the police came, and I talked to the officer. But I couldn't think straight, I just felt afraid for my children. He said they were in the hospital but I couldn't understand what had happened.

Prosecutor Cross-Examination:

Prosecutor: You seem to recall a lot about the conversation you had with your husband.
Mrs. Fujikawa: It was a very upsetting conversation.

Prosecutor: Were you at all surprised that he was going to leave you?
Mrs. Fujikawa: We had our differences, but I can't believe he would abandon us.

Prosecutor: But you knew he would, didn't you. You had fought many times before that day.
Mrs. Fujikawa: We would argue sometimes, but I knew he loved us. Our children always came first, no matter what our differences.

Prosecutor: Your children came first? Where was that instinct when you drove off the bridge? You didn't think about them then, did you?
Mrs. Fujikawa: I don't know what happened; I just blacked out.

Prosecutor: Do you recall telling your husband that he would regret his decision to leave?
Mrs. Fujikawa: I might have said that, but my memories are blurry.

Prosecutor: But it would be fair to say that you were angry with your husband?
Mrs. Fujikawa: I was angry, yes, but I loved him still. I wouldn't do anything to hurt him

Prosecutor: Don't you think losing his children has hurt him?
Mrs. Fujikwa: They're our children and they are what matter most in the whole world.
Prosecutor: But they're gone now, Mrs. Fujikawa. You made sure of that.

Prosecutor Closing Statement
[BOTH CONDITIONS]

The defendant, Mrs. Fujikawa, was in a deeply troubled marriage. When her husband called to tell her they would no longer be together, she had no cards left to play, no way to punish him. But there was one way, wasn't there? This is a case of evil and vengeance, ladies and gentlemen. Yuriko Fujikawa got back at her husband by killing his children. Did she want to take her own life? Perhaps she did, but that does not change the fact that she cut short two innocent lives. She could have pulled over, could have made sure they were safe. Most importantly, she didn't have to get into that car at all. She could have waited until she was calm enough to drive. But no, she was selfish and took the children with her off that bridge. The defense would like you to believe that she didn't mean to do it, that she did not realize that she was driving off of a bridge. That she loved her kids so much she would never hurt them. There's something off, about this story, ladies and gentlemen, because the fact is, she did hurt those children. You heard testimony that she would shout and get angry when things weren't going her way, that she vowed her husband would regret his decision. Doesn't this sound like a woman who was just plain angry? Angry at the man who was ruining her life, so she saw fit to ruin his. Does this sound like the act of a loving mother? Doesn't a loving mother protect her children at all costs? Not this mother, ladies and gentlemen. Mrs. Fujikawa is no loving mother, if she was then we would not be here talking about it. Her children would still be alive.

Defense's Closing Statement
[CULTURAL AUTOMATISM CONDITION ONLY]

This is not a case of evil. This is a case of sadness and despair. There's no one in this world more devastated about the event in question than my client. The prosecutor wants you to think that because Mrs. Fujikawa had a rocky marriage, that she would be angry enough to kill her own children. That, ladies and gentlemen, is a pretty big leap. I have to ask myself, how does a loving mother kill her children in cold blood? The short answer is, she doesn't. Mrs. Fujikawa devoted everything to her beautiful children, she was there every step of the way, and she wouldn't for a second abandon them the way her husband was abandoning the family. No, the facts don't fit, ladies and gentlemen. *You have to remember that Mrs. Fujikawa's culture is relevant in this case. In Japanese culture, divorce is a very serious issue, and it is thought to reflect failure on the part of the couple. Because of the implications of divorce in that culture, Mrs. Fujikawa was unable to cope with her husband's words and so she blacked out.* So what is a likely story? Imagine a mother who did everything right, who gave everything to her family and saw it fall apart in a split second. She did not get a chance to think about anything. In that split second she lost herself, and in that split second her body took control, not her mind. You heard expert testimony that my client experienced a trauma just as strong as a physical blow. The prosecution claims my client did not protect her children, but she did as soon as she got her mind back. She cried out for their safety, she thought only of them. She only ever thought of family. The prosecutor is trying to convince you my client is selfish. Does a selfish person drive off of a bridge? Does a person in their right mind do that? That sounds like quite a stretch, ladies and gentlemen. Do you know what's not a stretch? Thinking of a mother whose world was shattered in an instant when her family was torn apart.

Defense's Closing Statement
[STANDARD AUTOMATISM CONDITION ONLY]

This is not a case of evil. This is a case of sadness and despair. There's no one in this world more devastated about the event in question than my client. The prosecutor wants you to think that because Mrs. Fujikawa had a rocky marriage, that she would be angry enough to kill her own children. That, ladies and gentlemen, is a pretty big leap. I have to ask myself, how does a loving mother kill her children in cold blood? The short answer is, she doesn't. Mrs. Fujikawa devoted everything to her beautiful children, she was there every step of the way, and she wouldn't for a second abandon them the way her husband was abandoning the family. No, the facts don't fit, ladies and gentlemen. *You have to remember that to Mrs. Fujikawa, divorce is a very serious issue, and it is thought to reflect failure on the part of the couple. Because of the implications of divorce in Mrs. Fujikawa's world*, she was unable to cope with her husband's words and so she blacked out. So what is a likely story? Imagine a mother who did everything right, who gave everything to her family and saw it fall apart in a split second. She did not get a chance to think about anything. In that split second she lost herself, and in that split second her body took control, not her mind. You heard expert testimony that my client experienced a trauma just as strong as a physical blow. The prosecution claims my client did not protect her children, but she did as soon as she got her mind back. She cried out for their safety, she thought only of them. She only ever thought of family. The prosecutor is trying to convince you my client is selfish. Does a selfish person drive off of a bridge? Does a person in their right mind do that? That sounds like quite a stretch, ladies and gentlemen. Do you know what's not a stretch? Thinking of a mother whose world was shattered in an instant when her family was torn apart.

Prosecution Rebuttal
[BOTH CONDITIONS]

Yes, ladies and gentlemen. Her family was being torn apart. So what did she have to lose in taking those children with her into the water? Why don't you imagine a woman who, if family is so important, already knows she's lost everything. If she tried to take her own life, is it so hard to believe she would be prepared to kill her children, too? The protective, motherly instincts the defense would like you to believe in were simply absent. This is an angry person, a cold person who was tired of having no control over her marriage. Yes, ladies and gentlemen, her world was shattered, but does that mean it's O.K. to fail to protect your children? It is up to you, the jury, to hold Yuriko Fujikawa responsible for killing that wonderful girl and boy. This so-called "fugue" state is just an excuse, an excuse she needed only because she failed to kill herself too. You heard witnesses testify that Mrs. Fujikawa seemed distraught and confused. She was distraught and confused because she knew what she did was wrong, and she was the one who regretted it in the end.
