# Supplementary material for: Culture in the Courtroom: Ethnocentrism and Juror Decision-Making
Source: PLoS One. 2015 Sep 9;10(9):e0137799. doi: 10.1371/journal.pone.0137799 (PMC4564170; doi:10.1371/journal.pone.0137799)
Supplement: S2 Text — (PDF) [file pone.0137799.s003.pdf]

Male Defendant Trial Transcripts

Note: italics have been added to this document for emphasis of manipulated content, but were not italicized for participants

[BOTH CONDITIONS]

Background Information
Alleged Crime: Second Degree Murder
Victims: Mari Fujikawa, Kenichi Fujikawa
Defendant: Mr. Toshi Fujikawa D.O.B.: July 12th, 1979
Arrested: May 16th, 2012

Prosecution Opening Statement

The Prosecution will prove that Toshi Fujikawa drove his two children into a lake, intending to kill them. You will see that Mr. Fujikawa received news that his wife had an affair and was leaving him, and wanted to punish her in the only way that he knew how, by taking away her children forever. Even though the defendant himself went into the water with the children, he committed second-degree murder when he drove off that bridge knowing full well that the children would die. The Prosecution will provide a witness that saw Toshi Fujikawa drive into the lake that afternoon, show that he had motive to kill his children, and demonstrate that he was fully aware of his actions at the time of the crime. In short, we will prove beyond any reasonable doubt that Toshi Fujikawa is guilty of murder in the second degree. Toshi Fujikawa is a cold-blooded killer who took two innocent lives because he wanted revenge on his wife, plain and simple.

Defense Opening Statement

The Prosecution is going to try to spin a heartless tale to make you believe that my client is evil. They are going to try to piece together a questionable story that does not fit with my client's great love of his children, but instead use some pretty slippery arguments to convince you that he is a bad person. The problem with the Prosecution's story is that my client is not evil; he is a father who loved his family so much that when infidelity threatened to break it apart, he lost himself completely and blacked out. The Defense will show that the Prosecution's claim that my client meant to hurt those children he loved most out of anything in the world is baseless, and in fact it was that love of family that led to the severe psychological blow he suffered from hearing about his wife's affair. You will see that he did not commit this act voluntarily but instead lost control in those brief moments. Toshi Fujikawa is not a calculating killer, and you, the jury, will be the real villains if you send this man to jail.

Prosecution Witness, Andrew Morris
[BOTH CONDITIONS]

Prosecutor: What did you do on the afternoon of May 16th, 2012?
Morris: I went fishing at the lake.

Prosecutor: What did you see while you were at the lake?
Morris: I saw a car drive on to the bridge and start turning toward the edge. I didn't understand what they were doing, but then I saw the car go right off the bridge into the water.

Prosecutor: What did you do then?
Morris: I grabbed my cell phone and called 911, and asked them to send an ambulance and the police.

Prosecutor: Then what happened?
Morris: I had to try to help them, so I swam up to the front of the car, and managed to pull the man in the driver's seat out through the window. I brought him to the shore and he seemed to be alright.

Prosecutor: Did he say anything?
Morris: He kept saying his kids were in the car.

Prosecutor: Then what did you do?
Morris: I swam as fast as I could back to the car to help the children

Prosecutor: What happened next?
Morris: The police arrived and jumped in the water to help. We were able to pull the two children ashore.

Defense Cross-Examination:

Defense: Mr. Morris, did you ask my client if anyone else was in the car?
Morris: No, he just kept saying it when we got to the shore.

Defense: Did he say anything when you pulled him out of the car?
Morris: It all happened so fast and it was hard to hear anything while I was trying to keep us above water.

Defense: Is it possible my client was asking you to help his children first?
Morris: Sure, it's possible.

Defense: Did Mr. Fujikawa seem coherent to you?
Morris: He seemed very distraught and confused.

Prosecution Re-direct:

Prosecutor: So, you pulled Mr. Fujikawa to the shore and just moments after the incident he was coherent enough to ask you to help his children?
Morris: Yes, I guess that's right. At least, I understood what he was saying.

Prosecution Witness, Officer Scott Townsend
[BOTH CONDITIONS]

Prosecutor: Can you please state your name and occupation?
Townsend: My name is Officer Scott Townsend, I am a police officer and was the first to arrive on scene on the day in question. I was also the officer that arrested Toshi Fujikawa.

Prosecutor: What happened when you arrived on scene?
Townsend: When I arrived, I observed a man lying on the shore, and a man in the water struggling.

Prosecutor: Who did you identify the man on shore as?
Townsend: The defendant, Toshi Fujikawa.

Prosecutor: What did you do then?
Townsend: I got into the water to help the other man. We were able to pull the two children to the shore. At that time the paramedics attempted to resuscitate the children and transported them to the hospital.

Prosecutor: What happened after that?
Townsend: While the paramedics were attending to Mr. Fujikawa, I interviewed the witness, Andrew Morris.

Prosecutor: What did you find out from interviewing Mr. Morris?
Townsend: He indicated that he had seen the car drive off of the bridge into the water, and that the incident did not appear to be a simple car accident. At that time, since the children were in critical condition and there was evidence to suggest that he purposefully drove off of the bridge, not as a result of loss of control of the vehicle, I arrested Mr. Fujikawa.

Defense Cross-examination:

Defense: Officer Townsend, did you interview my client at the time of the event in question?
Townsend: Yes, I did.

Defense: And would you say that he was able to answer your questions?

Townsend: Mr. Fujikawa acted strangely and was not able to answer my questions coherently.

Prosecution Re-direct:

Prosecutor: Officer Townsend, did the defendant communicate to you at any time that he was aware that his children were in critical condition?
Townsend: He continued to ask about his children, if they were alive.

Prosecutor: So, just moments after the incident, when he was supposedly unaware of what was happening, he communicated that he knew the children were in danger, and that they might not live?
Townsend: Yes, that's correct.

Prosecution Witness, Mrs. Yuriko Fujikawa
[BOTH CONDITIONS]

Prosecutor: Could you please state your name and relationship to the defendant?
Mrs. Fujikawa: My name is Mrs. Yuriko Fujikawa; I am married to Toshi Fujikawa.

Prosecutor: Did you speak to the defendant on the day in question?
Mrs. Fujikawa: I called him that afternoon when he was picking the kids up from school.

Prosecutor: Why did you call your husband that afternoon?
Mrs. Fujikawa: I called to tell him that I could no longer stay in this marriage. I could no longer keep it a secret that I love another man. I planned to leave before he and the kids came home.

Prosecutor: How did he react to this news?
Mrs. Fujikawa: He sounded very angry. We had been fighting for some weeks before that conversation, and he would yell and curse at me every time.

Prosecutor: Can you recall what the defendant said?
Mrs. Fujikawa: He said something like "you'll regret this."

Prosecutor: How long did your conversation last on that particular occasion?
Mrs. Fujikawa: Only a few minutes, I knew if I stayed on the phone longer he would only yell and I had already made up my mind. So I hung up after just a few minutes.

Defense Cross-examination:

Defense: Mrs. Fujikawa, did you often pick the children up from school?
Mrs. Fujikawa: Toshi usually did that.

Defense: Would you say that your husband spent a lot of time with the children, took good care of them?
Mrs. Fujikawa: Yes, he did put a lot of time in to taking care of them.

Defense: And in all the times you claim to have fought with your husband, at any time did you observe him to take his anger out on the children?
Mrs. Fujikawa: No, he only got angry with me.

Defense Witness, Dr. Elliot Green
[STANDARD AUTOMATISM CONDITION ONLY]

Defense: Can you please state your name and occupation for the court?
Green: Yes. My name is Dr. Elliot Green, I am a psychiatrist working at the Health Science Center.

Defense: And what are your credentials?
Green: I have a medical degree and have been practicing psychiatry for 20 years now.

Defense: Have you spoken extensively with my client, Mr. Fujikawa?
Green: Yes. I have been meeting with Mr. Fujikawa once a week for the past month. We have discussed the incident in a lot of detail.

Defense: Could you describe to the court what you learned from meeting with Mr. Fujikawa?
Green: Certainly. He had communicated to me that he began to suspect his wife was being unfaithful, and that he felt very distraught over that. He indicated that family is the most important thing in his life and that he could not cope if he did not have them. \

Defense: And what about the day in question, did the two of you speak about that?
Green: Yes, we did. He described getting in to the car and blacking out after hearing his wife's words. The next memory he reported was feeling very disoriented surrounded by police officers. He remarked to me that at that time, he feared for his children.

Defense: Could you describe to the court what can happen when a person experiences a psychological trauma?
Green: Yes. Sometimes, when the mind receives information for which it is unprepared, a condition can occur in which there is loss of the usual integration of personal identity and memories, sensory and motor function. As such, there is a splitting of specific mental activities from the rest of conscious awareness. In less extreme cases, for example, we may drive to a destination while distracted, and not recall the actual drive.

Defense: In your opinion, is Mr. Fujikawa's experience consistent with such a state?
Green: Yes. Receiving such news as he did that his wife would be leaving would be much the same as receiving a physical blow to the head, except that it was a blow to the mind. Receiving such emotional news can leave one in a fugue state for several minutes, where continuing to drive is a very plausible event. *In Mr. Fujikawa's case, divorce*

seemed to him a very serious issue. Therefore, he likely reacted quite strongly to his wife's news.

Defense: Could you elaborate on what kind of experience could prompt such a severe reaction?
Green: Mr. Fujikawa talked at length about how much his family was central to his life. He often spoke of his willingness to do anything for the sake of family. *He indicated that for him, family always comes first, and he considers divorce to mean grave failure. Given how serious divorce is for him, it is quite plausible that he would be placed in such a dissociative state, unable to cope with the implications of the divorce.* He did not expect to receive support from extended family, and did not indicate he was aware of any social support programs.

Defense: In your opinion, is Mr. Fujikawa trying to mislead you into believing he experienced this trauma?
Green: No, I do not believe so. In my 20 years of practice, I have come into contact with a few cases of false claims of mental trauma and loss of control. Typically, such a person would have inconsistencies in their report or might provide unnecessary details of the event.

Prosecution Cross-examination:

Prosecutor: Dr. Green, is the area of dissociative states your area of expertise?
Green: No, I mostly treat patients with severe long-term mental disorders.

Prosecutor: Are you an expert in deception, Dr. Green?
Green: No, I am not. But I have many years experience treating real psychological issues.

Prosecutor: I see. You said that Mr. Fujikawa remarked that he was suspicious of his wife's infidelity in the time leading up to the crime.
Green: Yes, that's correct.

Prosecutor: So, isn't it possible that Mr. Fujikawa was very angry at his wife as a result?
Green: Yes, that's possible. But from my interviews with Mr. Fujikawa, I believe this saddened him more than anything.

Prosecutor: To your knowledge, had Mr. Fujikawa suffered a fugue state like this prior to the incident?

Green: No, not to my knowledge. This incident seemed to be particularly upsetting to him so as to create the dissociative state.

Prosecutor: So, no prior fugue states such as this, and he just happens to experience one when he breaks the law?
Green: He did not indicate he had suffered something like this before.

Prosecutor: Well that sounds pretty convenient for Mr. Fujikawa, doesn't it? So, to clarify, you do not typically treat patients who have had fugue states, and this was Mr. Fujikawa's first ever fugue state.
Green: I suppose that's true, yes.

Defense Witness, Dr. Elliot Green
[CULTURAL AUTOMATISM CONDITION ONLY]

Defense: Can you please state your name and occupation for the court?
Green: Yes. My name is Dr. Elliot Green, I am a psychiatrist working at the Health Science Center.

Defense: And what are your credentials?
Green: I have a medical degree and have been practicing psychiatry for 20 years now.

Defense: Have you spoken extensively with my client, Mr. Fujikawa?
Green: Yes. I have been meeting with Mr. Fujikawa once a week for the past month. We have discussed the incident in a lot of detail.

Defense: Could you describe to the court what you learned from meeting with Mr. Fujikawa?
Green: Certainly. He had communicated to me that he began to suspect his wife was being unfaithful, and that he felt very distraught over that. He indicated that family is the most important thing in his life and that he could not cope if he did not have them.

Defense: And what about the day in question, did the two of you speak about that?
Green: Yes, we did. He described getting in to the car and blacking out after hearing his wife's words. The next memory he reported was feeling very disoriented surrounded by police officers. He remarked to me that at that time, he feared for his children.

Defense: Could you describe to the court what can happen when a person experiences a psychological trauma?
Green: Yes. Sometimes, when the mind receives information for which it is unprepared, a condition can occur in which there is loss of the usual integration of personal identity and memories, sensory and motor function. As such, there is a splitting of specific mental activities from the rest of conscious awareness. In less extreme cases, for example, we may drive to a destination while distracted, and not recall the actual drive.

Defense: In your opinion, is Mr. Fujikawa's experience consistent with such a state?
Green: Yes. Receiving such news as he did that his wife would be leaving would be much the same as receiving a physical blow to the head, except that it was a blow to the mind. Receiving such emotional news can leave one in a fugue state for several minutes, where continuing to drive is a very plausible event. *In Japanese culture, divorce is a very serious issue. Therefore, a person from his culture will typically react quite strongly to such news.*

Defense: Could you elaborate on what kind of experience could prompt such a severe reaction?
Green: Mr. Fujikawa talked at length about how much his family was central to his life. He often spoke of his willingness to do anything for the sake of family. *In Japanese culture, family always comes first, and inability to keep the family together is considered a grave failure. Given how serious divorce is in his culture, and the intense shame that resulted for him, it is very plausible that he would be placed in such a dissociative state, unable to cope with the cultural implications of the divorce.* He would not likely receive support from extended family in this case, and would not necessarily be aware of social support programs here

Defense: In your opinion, is Mr. Fujikawa trying to mislead you into believing he experienced this trauma?
Green: No, I do not believe so. In my 20 years of practice, I have come into contact with a few cases of false claims of mental trauma and loss of control. Typically, such a person would have inconsistencies in their report or might provide unnecessary details of the event.

Prosecution Cross-examination:

Prosecutor: Dr. Green, is the area of dissociative states your area of expertise?
Green: No, I mostly treat patients with severe long-term mental disorders.

Prosecutor: Are you an expert in deception, Dr. Green?
Green: No, I am not. But I have many years experience treating real psychological issues.

Prosecutor: I see. You said that Mr. Fujikawa remarked that he was suspicious of his wife's infidelity in the time leading up to the crime.
Green: Yes, that's correct.

Prosecutor: So, isn't it possible that Mr. Fujikawa was very angry at his wife as a result?
Green: Yes, that's possible. But from my interviews with Mr. Fujikawa, I believe this saddened him more than anything.

Prosecutor: To your knowledge, had Mr. Fujikawa suffered a fugue state like this prior to the incident?
Green: No, not to my knowledge. This incident seemed to be particularly upsetting to him so as to create the dissociative state.

Prosecutor: So, no prior fugue states such as this, and he just happens to experience one when he breaks the law?
Green: He did not indicate he had suffered something like this before.

Prosecutor: Well that sounds pretty convenient for Mr. Fujikawa, doesn't it? So, to clarify, you do not typically treat patients who have had fugue states, and this was Mr. Fujikawa's first ever fugue state.
Green: I suppose that's true, yes.

Defense Witness, Toshi Fujikawa
[BOTH CONDITIONS]

Defense: What happened on the afternoon of May 16th?
Mr. Fujikawa: I picked up my children from school. My wife called me as I was helping them into the car.

Defense: Were you happy to see them?
Mr. Fujikawa: Yes of course. They were happy to see me too. I picked them up every day, and it was always the best part of my day.

Defense: I know this is very difficult, but can you tell the court what your wife said when you spoke with her?
Mr. Fujikawa: I think she said…that she would be leaving that day. Leaving the family.

Defense: How did that make you feel, when she told you that?
Mr. Fujikawa: It was very difficult to hear. My family is my life. Family is all that matters. To abandon your family, it's unthinkable.

Defense: I know how much you loved your children, Mr. Fujikawa. Can you try to describe what happened after that?
Mr. Fujikawa: No, I can't. I just can't quite understand what happened. I was talking with my wife, and the next thing I knew I was struggling to breathe and there were people all around me.

Defense: What else can you recall from the drive home?
Mr. Fujikawa: I don't know. I felt sick, I don't know what happened. I think the police came, and I talked to the officer. But I couldn't think straight, I just felt afraid for my children. He said they were in the hospital but I couldn't understand what had happened.

Prosecutor Cross-Examination:

Prosecutor: You seem to recall a lot about the conversation you had with your wife.
Mr. Fujikawa: It was a very upsetting conversation.

Prosecutor: Were you at all surprised that she was going to leave you?
Mr. Fujikawa: We had our differences, but I can't believe she would abandon us.

Prosecutor: But you knew she would, didn't you. You had fought many times before that day.
Mr. Fujikawa: We would argue sometimes, but I knew she loved us. Our children always came first, no matter what our differences.

Prosecutor: Your children came first? Where was that instinct when you drove off the bridge? You didn't think about them then, did you?
Mr. Fujikawa: I don't know what happened; I just blacked out.

Prosecutor: Do you recall telling your wife that she would regret her decision to leave?
Mr. Fujikawa: I might have said that, but my memories are blurry.

Prosecutor: But it would be fair to say that you were angry with your wife?
Mr. Fujikawa: I was angry, yes, but I loved her still. I wouldn't do anything to hurt her.

Prosecutor: Don't you think losing her children has hurt her?
Mr. Fujikawa: They're our children and they are what matter most in the whole world.
Prosecutor: But they're gone now, Mr. Fujikawa. You made sure of that.

Prosecutor Closing Statement
[BOTH CONDITIONS]

The defendant, Mr. Fujikawa, was in a deeply troubled marriage. When his wife called to tell him they would no longer be together, he had no cards left to play, no way to punish her. But there was one way, wasn't there? This is a case of evil and vengeance, ladies and gentlemen. Toshi Fujikawa got back at his wife by killing her children. Did he want to take his own life? Perhaps he did, but that does not change the fact that he cut short two innocent lives. He could have pulled over, could have made sure they were safe. Most importantly, he didn't have to get into that car at all. He could have waited until he was calm enough to drive. But no, he was selfish and took the children with him off that bridge. The defense would like you to believe that he didn't mean to do it, that he did not realize that he was driving off of a bridge. That he loved his kids so much he would never hurt them. There's something off, about this story, ladies and gentlemen, because the fact is, he did hurt those children. You heard testimony that he would shout and get angry when things weren't going his way, and that he vowed his wife would regret her decision. Doesn't this sound like a man who was just plain angry? Angry at the woman who was ruining his life, so he saw fit to ruin hers. Does this sound like the act of a loving father? Doesn't a loving father protect his children at all costs? Not this father, ladies and gentlemen. Mr. Fujikawa is no loving father, if he was then we would not be here talking about it. His children would still be alive.

Defense's Closing Statement
[STANDARD AUTOMATISM CONDITION ONLY]

This is not a case of evil. This is a case of sadness and despair. There's no one in this world more devastated about the event in question than my client. The prosecutor wants you to think that because Mr. Fujikawa had a rocky marriage, that he would be angry enough to kill his own children. That, ladies and gentlemen, is a pretty big leap. I have to ask myself, how does a loving father kill his children in cold blood? The short answer is, he doesn't. Mr. Fujikawa devoted everything to his beautiful children, he was there every step of the way, and he wouldn't for a second abandon them the way his wife was abandoning the family. No, the facts don't fit, ladies and gentlemen. *You have to remember that to Mr. Fujikawa, divorce is a very serious issue, and it is thought to reflect failure on the part of the couple. Because of the implications of divorce in Mr. Fujikawa's world*, he was unable to cope with his wife's words and so he blacked out. So what is a likely story? Imagine a father who did everything right, who gave everything to his family and saw it fall apart in a split second. He did not get a chance to think about anything. In that split second he lost himself, and in that split second his body took control, not his mind. You heard expert testimony that my client experienced a trauma just as strong as a physical blow. The prosecution claims my client did not protect his children, but he did as soon as he got his mind back. He cried out for their safety, he thought only of them. He only ever thought of family. The prosecutor is trying to convince you my client is selfish. Does a selfish person drive off of a bridge? Does a person in their right mind do that? That sounds like quite a stretch, ladies and gentlemen. Do you know what's not a stretch? Thinking of a father whose world was shattered in an instant when his family was torn apart.

Defense's Closing Statement
[CULTURAL AUTOMATISM CONDITION ONLY]

This is not a case of evil. This is a case of sadness and despair. There's no one in this world more devastated about the event in question than my client. The prosecutor wants you to think that because Mr. Fujikawa had a rocky marriage, that he would be angry enough to kill his own children. That, ladies and gentlemen, is a pretty big leap. I have to ask myself, how does a loving father kill his children in cold blood? The short answer is, he doesn't. Mr. Fujikawa devoted everything to his beautiful children, he was there every step of the way, and he wouldn't for a second abandon them the way his wife was abandoning the family. No, the facts don't fit, ladies and gentlemen. *You have to remember that Mr. Fujikawa's culture is relevant in this case. In Japanese culture, divorce is a very serious issue, and it is thought to reflect failure on the part of the couple. Because of the implications of divorce in that culture, Mr. Fujikawa was unable to cope with his wife's words and so he blacked out.* So what is a likely story? Imagine a father who did everything right, who gave everything to his family and saw it fall apart in a split second. He did not get a chance to think about anything. In that split second he lost himself, and in that split second his body took control, not his mind. You heard expert testimony that my client experienced a trauma just as strong as a physical blow. The prosecution claims my client did not protect his children, but he did as soon as he got his mind back. He cried out for their safety, he thought only of them. He only ever thought of family. The prosecutor is trying to convince you my client is selfish. Does a selfish person drive off of a bridge? Does a person in their right mind do that? That sounds like quite a stretch, ladies and gentlemen. Do you know what's not a stretch? Thinking of a father whose world was shattered in an instant when his family was torn apart.

Prosecution Rebuttal
[BOTH CONDITIONS]

Yes, ladies and gentlemen. His family was being torn apart. So what did he have to lose in taking those children with him into the water? Why don't you imagine a man who, if family is so important, already knows he's lost everything. If he tried to take his own life, is it so hard to believe he would be prepared to kill his children, too? The protective, fatherly instincts the defense would like you to believe in were simply absent. This is an angry person, a cold person who was tired of having no control over his marriage. Yes, ladies and gentlemen, his world was shattered, but does that mean it's O.K. to fail to protect your children? It is up to you, the jury, to hold Toshi Fujikawa responsible for killing that wonderful girl and boy. This so-called "fugue" state is just an excuse, an excuse he needed only because he failed to kill himself too. You heard witnesses testify that Mr. Fujikawa seemed distraught and confused. He was distraught and confused because he knew what he did was wrong, and he was the one who regretted it in the end.
